# 伊立替康联合奈达铂对比联合顺铂二线治疗复发或难治性小细胞肺癌的疗效及毒副作用回顾分析

**DOI:** 10.3779/j.issn.1009-3419.2013.09.06

**Published:** 2013-09-20

**Authors:** 舒飞 于, 燕 王, 兴胜 胡, 宏羽 王, 学志 郝, 建萍 许, 峻岭 李, 湘茹 张, 远凯 石

**Affiliations:** 100021 北京，中国医学科学院肿瘤医院肿瘤内科 Department of Medical Oncology, Cancer Institute (Hospital), Chinese Academy of Medical Sciences and Peking Union Medical College, Beijing 100021, China

**Keywords:** 肺肿瘤, 伊立替康, 铂类, 复发, Lung neoplasms, Irinotecan, Platinum, Relapsed

## Abstract

**背景与目的:**

对初治进展或复发的小细胞肺癌，目前尚无标准的二线方案，本研究旨在比较伊立替康联合奈达铂或联合顺铂治疗敏感复发或难治性小细胞肺癌的疗效和安全性。

**方法:**

回顾了中国医学科学院肿瘤医院2009年4月-2012年4月诊治的1, 140例小细胞肺癌患者，筛选二线接受伊立替康联合奈达铂（IN）或伊立替康联合顺铂（IC）方案化疗的患者进行分析。

**结果:**

入组的54例患者中，中位无进展生存时间（progression free survival, PFS）为4.9个月，中位总生存时间（overall survival, OS）为13.3个月，IC组的PFS为4.3个月，IN组的PFS为5.4个月（*P*=0.465）。两组OS分别为13.3个月和14.3个月（*P*=0.704）。对生存时间的*Cox*多因素分析显示：二线治疗前的PS评分（*P*=0.003）、二线治疗前的转移部位个数（*P*=0.023）、接受化疗的周期数（*P*=0.003）是独立预后因素。整体的不良反应可耐受，IN组血液学毒性较重，IC组腹泻发生率较高，但均无统计学意义。

**结论:**

伊立替康联合铂类是对于敏感复发和难治性小细胞肺癌有效且耐受性好的方案，伊立替康联合奈达铂在疗效及安全性方面都不劣于其联合顺铂。

## Abstract (en)

**Background and objective:**

At present no standard second-line combination has been established for recurrent small cell lung cancer (SCLC). Therefore we evaluate the efficacy and safety of irinotecan in combination with nedaplatin/cisplatin against refractory or relapsed small cell lung cancer.

**Methods:**

In this retrospective study, we analyzed the data of 1, 140 patients who diagnosed small cell lung cancer at our hospital from April 2009 to April 2012. Of all the patients, 34 patients were treated with irinotecan and nedaplatin (irinotecan 60 mg/m^2^ on days 1, 8 nedaplatin 85 mg/m^2^ day 1, every 3 weeks), and 20 patients were treated with irinotecan and cisplatin (irinotecan 60 mg/m^2^ on days 1, 8 cisplatin 75 mg/m^2^ day 1, every 3 weeks) as the second-line treatment. Prognostic factors of overall survival (OS) were estimated by *Kaplan-Meier* and *Cox*'s Regression-proportional hazards model.

**Results:**

Of all the 54 eligible patients, median progression free survival (PFS) was 4.9 months, and median OS was 13.3 months. Median PFS was 5.4 months for irinotecan plus nedaplatin (IN) and 4.9 months for irinotecan plus cisplatin (IC), respectively (*P*=0.465). Median OS was 14.3 months and 13.3 months, respectively (*P*=0.704). In multivariate analysis, ECOG PS, number of metastases and cycles of chemotherapy were independent prognostic factors. The toxicities were mild, while toxicity profile was slightly different for each of the arms: hematologic toxicity was higher in IN group, and diarrhea was higher in IC group.

**Conclusion:**

Irinotecan plus platinum is effective and tolerable for refractory and relapsed small cell lung cancer. Irinotecan plus nedaplatin is non-inferior to irinotecan plus cisplatin in terms of efficacy and safety.

小细胞肺癌约占肺癌的15%^[1]^，小细胞肺癌的一线治疗疗效明显^[2]^。但是绝大多数患者都会在随后的2年内复发，局限期患者的2年复发率为75%，而广泛期患者的2年复发率接近100%^[3]^。多数复发的小细胞肺癌患者体力状态迅速恶化，平均生存时间仅有2个月-4个月^[4]^。但是也有许多患者体力评分和器官的功能依旧良好，仍有机会可以接受二线治疗。目前NCCN指南推荐的二线治疗小细胞肺癌的方案有效率多在10%-20%，所以寻找更有效的方案迫在眉睫。

伊立替康是水溶性的喜树碱类的化合物，是拓扑异构酶Ⅰ抑制剂，可以干扰DNA的合成和影响有丝分裂。两个日本的小样本的临床实验^[5, 6]^中，单药伊立替康治疗难治性小细胞肺癌都取得了很好的疗效，有效率分别为50%和47%。

在一项日本的Ⅲ期临床试验（JCOG9511）^[7]^中，伊立替康联合顺铂组与足叶乙甙联合顺铂组相比明显提高了中位生存时间（12.8个月*vs* 9.4个月）和1年生存率（58.4% *vs* 37.7%）。奈达铂是顺铂的类似物。其肾毒性和神经毒性都比顺铂低很多，并与其他铂类药物无完全交叉耐药性^[8, 9]^。在体外实验中，伊立替康与奈达铂联合同伊立替康与顺铂联合一样具有协同作用，与奈达铂联合可使伊立替康的活性形式7-乙烷基-10-羟基喜树碱（SN-38）的拓扑异构酶Ⅰ的抑制活性增加10倍^[10]^。所以伊立替康联合奈达铂是治疗小细胞肺癌的一个很有前景的方案。本实验旨在评价伊立替康联合铂类是否为治疗复发的小细胞肺癌的可选方案，并探索性研究伊立替康与奈达铂联合是伊立替康与顺铂联合的可行的替代方案。

## 1 资料与方法

### 1.1 临床资料

病例入选标准：①中国医学科学院肿瘤医院经病理或细胞学确诊小细胞肺癌的患者；②影像学资料提示一线治疗中进展或治疗后复发；③一线治疗失败后接受至少1个周期伊立替康联合顺铂（IC）或伊立替康联合奈达铂（IN）方案化疗。排除标准：①不适合接受化疗的患者；②慢性炎性肠病和/或肠梗阻的患者；③血清总胆红素≥1.5 ULN的患者；④中性粒细胞计数绝对值（ANC）≤1, 500/μL的患者；⑤青光眼患者。

我们回顾了本院2009年4月-2012年4月收治的所有小细胞肺癌患者1, 140例，从中挑选一线治疗失败后使用伊立替康联合顺铂（IC）或伊立替康联合奈达铂（IN）方案化疗且病例记录完整者，共入组54例，中位年龄57岁，其中采用伊立替康联合顺铂方案的患者20例，采用伊立替康联合奈达铂方案的患者34例，两组患者的人口统计学特征相似（表 1）。

**1:** 患者临床特征 Clinical characteristics of included patients

Characteristics	Irinotecan+Nedaplantin (*n*=34)	Irinotecan+Cisplantin (*n*=20)
Age (yr)		
< 65	30 (88.2%)	17 (85.0%)
≥65	4 (11.8%)	3 (15.0%)
Gender		
Male	28 (82.4%)	16 (80.0%)
Female	6 (17.6%)	4 (20.0%)
Stage		
Limited	12 (35.3%)	5 (25.0%)
Extensive	22 (64.7%)	15 (75.0%)
Lung metastasis		
Yes	9 (26.5%)	5 (25.0%)
No	25 (73.5%)	15 (75.0%)
Bone metastasis		
Yes	9 (26.5%)	7 (35.0%)
No	25 (73.5%)	13 (65.0%)
Liver metastasis		
Yes	7 (20.6%)	3 (15.0%)
No	27 (79.4%)	17 (85.0%)
Brain metastasis		
Yes	15 (44.1%)	6 (30.0%)
No	19 (55.9%)	14 (70.0%)
Treatment-interval		
< 90 d	12 (35.3%)	6 (30.0%)
90 d-180 d	18 (52.9%)	9 (45.0%)
> 180 d	4 (11.8%)	5 (25.0%)

### 1.2 治疗方案

伊立替康联合奈达铂：伊立替康60 mg/m^2^，静脉滴注，第1, 8天+奈达铂85 mg/m^2^，静脉滴注，第2天/21天为1周期；伊立替康联合顺铂：伊立替康60 mg/m^2^，静脉滴注，第1, 8天+顺铂75 mg/m^2^，静脉滴注，第1天/21天为1周期。不超过6周期。

### 1.3 毒性评价和疗效评估

患者每个周期进行不良反应的评价，根据美国国立癌症研究所普通毒性分级3.0进行毒性反应分级。依据实体肿瘤疗效评价标准（Response Evaluation Criteria in Solid Tumors, RECIST）1.0每2个周期进行影像学检查评价疗效。分为完全缓解（complete response, CR），部分缓解（partial response, PR），疾病稳定（stable disease, SD）和疾病进展（progressive disease, PD），其中CR和PR至少持续4周。无进展生存时间（progression free survival, PFS）定义为从伊立替康联合铂类治疗开始至疾病进展的时间或死亡时间。总生存时间（overall survival, OS）定义为从伊立替康联合铂类治疗开始至患者死亡或末次随访的时间。敏感复发定义为从初始治疗结束时间至肿瘤进展时间超过90天；初始治疗不敏感，或治疗后90天内复发称为难治性复发。

### 1.4 随访

治疗结束后，每2个月进行1次随访，随访截止日期2012年12月30日。从二线治疗开始的中位随访时间为9.6个月。无患者失访。

### 1.5 统计学方法

应用SPSS 18.0软件进行统计分析。单因素及多因素分析采用*Cox*回归，PFS和OS生存分析采用*Kaplan-Meier*法，*P* < 0.05为差异有统计学意义。

## 2 结果

入组的54例患者（两组患者的临床特征见表 1），截止至2012年12月30日，中位随访时间为9.6个月。IC组的中位化疗周期数为3（1个-4个周期），其中4例患者减量1次；IN组中位化疗周期数为4（1个-6个周期），其中7例患者减量1次，1例患者减量2次。IC组出现1例治疗相关性死亡。

### 2.1 近期疗效和生存结果

入组的54例患者，无失访患者，49例患者可评价近期疗效。全部患者的中位PFS为4.9个月，中位OS为13.3个月。IC组的疾病缓解率（resopnse rate, RR）为33.3%，IN组的RR为29%（*P*=0.759）。两组的PFS及OS均无统计学差异，IC组的PFS为4.3个月，OS为13.3个月；IN组的PFS为5.4个月，OS为14.3个月（图 1，图 2）。IN组的6个月生存率为70.6%，1年生存率为26.5%；IC组的6个月生存率为70.0%，1年生存率为45%。敏感复发的患者中位生存时间为13.4个月，而难治性患者的中位生存时间为10.4个月（*P*=0.37）。敏感复发患者与难治性患者二线治疗的有效率分别为31.3%和30.3%（*P* > 0.999）。

**1:**
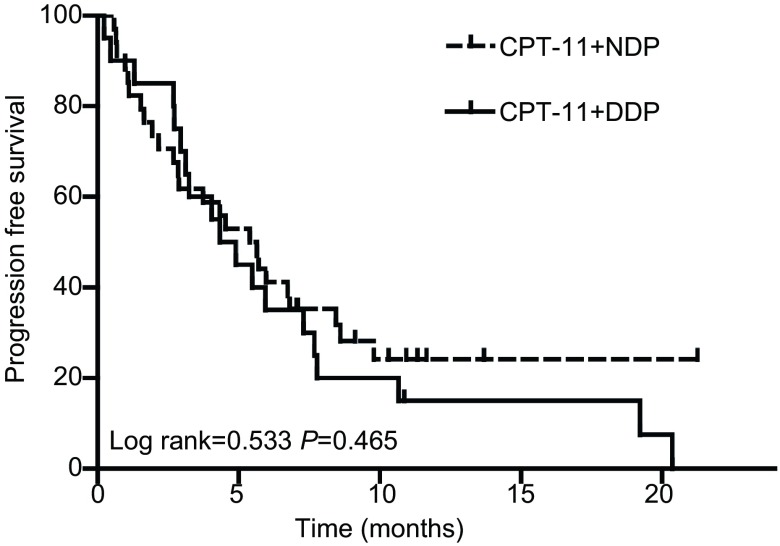
所有患者无进展生存期的*Kaplan-Meier*曲线 *Kaplan-Meier* curve of progression free survival (PFS) for all the patients

**2:**
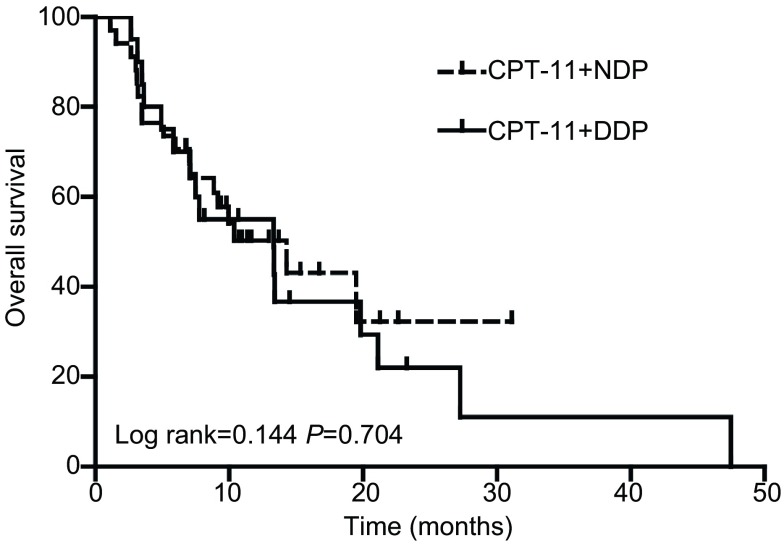
所有患者总生存的*Kaplan-Meier*曲线 *Kaplan-Meier* curve of overall survival (OS) for all the patients

### 2.2 不良反应

无论是血液学毒性还是非血液学毒性的发生率，两组均无统计学差别。但3度-4度的不良反应中，IN方案的血小板减少的发生率高于IC方案（*P*=0.174），但两组各有1例患者接受血小板输注治疗。而IC组的严重恶心呕吐和迟发型腹泻的发生率均高于IN组，*P*值均为0.133（表 2）。两组各有1例患者在治疗期间死亡。

**2:** 两组3度-4度不良反应 3-4° adverse events of two groups

	Irinotecan+Nedaplatin	Irinotecan+Cisplatin	*P*
Neutropenia	17 (50.0%)	7 (35.0%)	0.396
Thrombocytopenia	10 (29.4%)	2 (10.0%)	0.174
Anemia	7 (20.6%)	2 (10.0%)	0.458
Nausea & Vomiting	0 (0.0%)	2 (3.7%)	0.133
Diarrhea	0 (0.0%)	2 (3.7%)	0.133

### 2.3 影响PFS和OS的单因素和多因素分析

对全部患者的确诊分期（局限期*vs*广泛期）、性别（男*vs*女）、年龄（< 65岁*vs* ≥65岁）、二线治疗前是否出现肺转移（有*vs*无）、二线治疗前是否出现肝转移（有*vs*无）、二线治疗前是否出现脑转移（有*vs*无）、二线治疗前是否出现骨转移（有*vs*无）、二线治疗前是否出现肾上腺转移（有*vs*无）、二线治疗前的转移部位（单个*vs*多个）、接受含伊立替康方案治疗的周期数（1个-3个*vs* 4个-6个）、初始治疗时是否加入放疗（有*vs*无）、初始治疗结束至疾病复发的时间（< 90天*vs* 90天-180天*vs* > 180天）、初始治疗失败后复发的形式（局部复发*vs*远处转移）、接受二线治疗前的PS评分等可能影响PFS和OS的因素先进行单因素分析，根据单因素分析的结果选择预后因素进行*Cox*多因素回归分析。结果发现：接受二线治疗前的PS评分（*P*=0.043）是伊立替康联合铂类化疗的无进展生存时间的独立预后因素；接受二线治疗前的PS评分（*P*=0.003）、二线治疗前的转移部位个数（*P*=0.023）、接受含伊立替康方案治疗的周期数（*P*=0.003）是生存时间的独立预后因素（表 3）。

**3:** *Cox*多因素分析 Prognostic factors of survival in *Cox* progression analysis

Variables	*β*	SE	*P*	HR	95%CI for HR
NSE (normal *vs* abnormal)	0.066	0.559	0.906	1.068	0.357-3.196
Relapse intervals	0.260	0.607	0.668	1.297	0.395-4.258
No. of cycles (1-3 *vs* 4-6)	-1.749	0.586	0.003	0.174	0.055-0.549
Metastases (single *vs* multiple)	0.888	0.390	0.023	2.429	1.131-5.217
ECOG PS	1.269	0.420	0.003	3.557	1.562-8.099
Liver metastasis (Yes *vs* No)	-0.621	0.668	0.352	0.537	0.145-1.988
ECOG PS: Eastern Cooperative Oncology Group performance status					

## 3 讨论

小细胞肺癌是一种恶性程度极高的肿瘤，初治时对化疗敏感，局限期小细胞肺癌化疗的有效率为85%-95%，广泛期小细胞肺癌的有效率为60%-80%^[11]^，但小细胞肺癌缓解期很短，多数患者一线治疗后迅速复发。而且对于复发的患者，化疗的效果都不明显。因此NCCN指南首先推荐复发的小细胞肺癌患者参加临床实验。既往的文献报道，对于治疗后的小细胞肺癌患者，如果化疗的有效率超过10%，则认为化疗有效^[12]^。目前NCCN指南中推荐的方案主要来自于Ⅱ期临床试验的结果，一项临床实验显示接受多西他赛治疗的28例患者有效率为25%^[13]^，吉西他滨单药治疗化疗后的小细胞肺癌患者的有效率为11.9% ^[14]^。长春瑞滨单药在两项针对敏感复发患者的临床实验中，有效率分别为12%和16%^[15, 16]^。一项Ⅱ期临床实验的结果提示紫杉醇周疗方案的有效率为23.8%^[17]^。2012年Pietanza等^[18]^报道了替莫唑胺治疗复发或难治的小细胞肺癌，有效率为22%，中位生存时间达5.8个月。唯一一项针对复发患者的Ⅲ期临床实验^[19]^证明一线化疗结束后60天以上复发的小细胞肺癌患者（敏感复发）的中，拓扑替康单药的疗效与环磷酰胺+长春新碱+多柔比星三药联合的方案等效：有效率24.3% *vs*. 18.3%，复发后的中位生存时间25周*vs*. 24.7周，并且拓扑替康单药可达到更好的生活质量评分和症状缓解，基于此临床试验的结果，NCCN指南推荐无病进展时间 > 2个-3个月的患者选择拓扑替康方案化疗（1类证据），美国食品药品监督管理局也批准拓扑替康治疗复发的小细胞肺癌的适应症。但是单药拓扑替康的疗效并不理想。相比之下，含伊立替康的方案有较强优势，两个早期的日本的小样本的临床实验中，单药伊立替康治疗难治性小细胞肺癌都取得了很好的疗效，有效率分别为50%和47%^[5, 6]^。但这两项研究的例数较少，均仅有十几例，有效率也较其他单药二线治疗复发的小细胞肺癌的有效率高，另一项近期的土耳其的实验，入组了46例复发的小细胞肺癌患者，接受伊立替康单药治疗，有效率为17.5%，结果似乎更为可信^[20]^。日本的Ⅲ期临床试验（JCOG9511）也证实在广泛期小细胞肺癌患者中一线使用伊立替康联合顺铂方案优于足叶乙甙联合顺铂方案^[7]^。本实验的结果显示伊立替康联合铂类方案治疗复发或难治性小细胞肺癌患者的有效率为30.6%，与其他单药方案相比有效率明显提高。本实验中伊立替康联合铂类方案治疗敏感复发或难治性小细胞肺癌的中位PFS为4.9个月，中位的OS达到了13.3个月，也明显优于其他单药方案。这一优势可能与入组患者的选择有关，本实验中患者的一般状态较好，大多数患者开始含伊立替康方案治疗前的PS评分为0分-1分，可以经受较强的治疗，并且这种近期的疗效可以转化为生存获益。Song等^[21]^回顾性分析了193例小细胞肺癌二线治疗的患者，发现采用联合化疗较单药化疗有更高的缓解率（25.4% *vs* 9.1%, *P*=0.012）和更长的PFS（3.80个月*vs* 2.13个月，*P*=0.001）。由此，对于PS评分低、器官功能好的患者，采用联合方案符合伦理要求。另外本实验中，虽然敏感复发和耐药复发的患者的中位生存时间相差3个月，但是两组的近期疗效和远期疗效均无统计学差别，可能与样本量较小有关。

本实验中，IN组与IC组在近期疗效及远期疗效上均无差异。2013年发表的一篇日本的伊立替康联合奈达铂一线治疗广泛期小细胞肺癌的Ⅱ期临床试验文章中ORR为100%，中位PFS及中位OS分别为6.6个月和16个月^[22]^。同既往伊立替康联合顺铂方案的临床数据比较，总生存时间相似^[7]^。这也提示伊立替康联合奈达铂方案的疗效不劣于伊立替康联合顺铂方案。

IC组的严重消化道反应发生率高于IN组，但无统计学差异（*P*=0.133）；IN组的3度-4度血小板降低较重（*P*=0.174），但大多数可以经过白介素-11的治疗得以纠正，不影响后续治疗。两组各有1例患者在治疗期间死亡。IN组1例患者化疗中出现脑梗塞，并死于脑梗塞。该患者既往吸烟30包年，高血压病史8年，糖尿病史10年，且行全脑放疗后，有脑梗塞发生的高危因素，考虑死亡与化疗无关。IC组1例患者出现肠梗阻导致死亡，考虑为治疗相关性死亡，既往的SWOG0124实验中发现：*UGT1A1*和*ABCB1*基因都与伊立替康的毒性相关^[23]^，目前此基因检测在国内临床实践中并不普及，可鼓励在日后的临床工作中通过基因检测排除高危患者，保护患者利益。IC组和IN组无论在疗效和安全性方面都相似，提示IN方案是治疗复发的小细胞肺癌患者的有效方案。另外，多数患者在一线治疗中采用了标准的足叶乙甙联合顺铂方案，因顺铂的剂量限制性的神经毒性和中性粒细胞降低，二线时继续采用含顺铂的方案风险较高，并且容易出现耐药，采用IN方案可避免此类风险。

另一方面，本实验中有48.1%的患者在二线治疗进展后接受了三线治疗，接受了三线化疗的患者生存时间明显长于未接受三线治疗的患者（*P*=0.033）。在接受三线治疗的患者中，19例（73.1%）接受了联合方案化疗，其中7例（26.9%）患者接受了环磷酰胺+长春新碱+表阿霉素的方案的化疗，7例（26.9%）患者接受了紫杉醇+异环磷酰胺方案化疗。可见二线伊立替康联合铂类治疗失败的患者部分保持良好的体力状态，可以从后续治疗中获益。

对于OS的*Cox*多因素分析的结果显示：接受二线治疗前的PS评分（*P*=0.003）、二线治疗前的转移部位个数（*P*=0.023）、接受含伊立替康方案治疗的周期数（*P*=0.003）是总生存的独立预后因素。患者器官转移的部位个数与预后相关，而不同的转移部位对生存时间无明显影响。接受伊立替康联合铂类的化疗周期较多的患者，生存期也更长，说明患者从二线治疗的获益可以转化为生存获益。除了肿瘤本身的生物学行为及患者的一般状态，尚未发现其他强有力的预后因子。本实验中57.4%的患者在初始治疗中接受了胸部放疗，多因素分析显示是否接受胸部放疗对二线治疗开始后的生存时间无影响（*P*=0.450）。

本实验中伊立替康联合铂类的方案与其他单药方案的实验结果相比，提高了有效率和生存时间。对于一般状态好的患者，选择联合方案似乎有更多的生存获益。而伊立替康与奈达铂联合无论从疗效和安全性都与含顺铂方案相似，可作为二线治疗的备选方案。本文为回顾性分析，病例数目有限，联合方案的疗效和不良反应结果尚需前瞻性的临床试验证实。
